# Outcome of the cementless Taperloc stem

**DOI:** 10.3109/17453674.2011.627494

**Published:** 2011-11-24

**Authors:** Jeffrey R. McLaughlin

**Affiliations:** Medical Director Kennedy Center for the Hip and Knee, Mercy Medical Center2700 W Ninth Ave Suite 125, Oshkosh, WI 54904mjohnson@KCHipandknee.com

Sir—I write this letter to point out a grave error in the paper entitled “Outcome of the cementless Taperloc stem: a comprehensive literature review including arthroplasty register data” (Labek et al. 2011).

The paper incorrectly identifies Richard Rothman as the designer of the Taperloc femoral component. In fact, the developer of the Taperloc stem was William Kennedy at the hip and knee center in Oshkosh, WI. Therefore, the paper's conclusion that “the excellent results published by the developer's clinic are generally not reproducible by other surgeons” is invalid. The 4 articles authored by McLaughlin and Lee referenced in this paper present the rate of revision of the Taperloc stem from the center where the Taperloc originated. The only 4 articles authored by a Taperloc developer are the 4 from McLaughlin et al. The differences in revision rates for the Taperloc as published by McLaughlin and Lee versus the other (non-developing) authors, as well as for McLaughlin and Lee versus the registry data are both within a factor 3 and thus are not clinically relevant per the criterion used by Labek et al. to define outlier datasets. If this paper had correctly identified the designer of the Taperloc their conclusion would have been: Studies originating from the center where the Taperloc stem was developed accurately reflect the data published from other centers as well as registry data.
