# Supplementary material for: Outcome of the cementless Taperloc stem
Source: Acta Orthop. 2011 Nov 24;82(5):633–4. doi: 10.3109/17453674.2011.627494 (PMC3242964; doi:10.3109/17453674.2011.627494)
Supplement: Related texts

# Letter: Outcome of the cementless Taperloc stem

**Published:** 2011-11-24

**Authors:** Gerold Labek

**Affiliations:** Department of Orthopaedics, Innsbruck Medical University, Innsbruck, Austriagerold.labek@efort.org

*Sir—*The study had clear inclusion criteria for the identification of inventors, which are mentioned in the paper according to regular scientific procedures: (Co-)Authorship and reference to the affiliation by the manufacturer of the implant and/or in peer reviewed, Medline-listed publications.

We were aware of Dr. Kennedy's contribution through a Biomet/Rothman paper from 1995, which indicates Dr. Kennedy's affiliation as “Kennedy Center for Hip and Knee, Neenah, WI”.

Since Dr. Kennedy was not listed as (co-)author of the papers identified, since the affiliation was not identical, and since Biomet did not make reference to Dr. Kennedy or Dr. McLaughlin on their website or documents, we were not able to include these papers according to the inclusion criteria.

Dr. Rothman in contrast was mentioned as inventor by the manufacturer. Biomet deleted these references from their web page (without substitution by Dr. Kennedy or Dr. McLaughlin, who claims to be inventor on his web page, too). I have no detailed information whether this was due to the fact that we raised critical questions at that time, or due to the fact that Dr. Rothman was centrally involved in the design of a very similar stem (Accolade) for a Biomet competitor (Stryker).

In his letter (and on the website of the Kennedy Center) Dr. McLaughlin speaks of a close cooperation with Dr. Kennedy. The average follow-up of the patients included in the publications referenced in our paper is longer than Dr. McLaughlin's affiliation to the Kennedy Center. In my view it is remarkable that Dr. Kennedy was not co-author of the publications although Dr. McLaughlin claims the contribution now.

For these reasons I cannot accept the statement that publications by McLaughlin and Lee are the only articles “authored by the Taperloc developers”, the main statement of the letter.

I would like to propose to respect the basic attitude at least in European Journals not to mix up marketing and science.

Some final comments to Dr. McLaughlin:

I have checked the Kennedy Center's website.

My congratulations on the outcome results published there (<1% Revision rate for Taperloc after 26 years; A ratio of 15 to Register data and a ratio of almost 10 to the previous papers published by him). Although the published revision rate only seems to include aseptic loosening, a remarkable difference remains since in worldwide Register data more than 55% of all revisions in THA are due to aseptic loosening.

The alternative hypothesis (Oshkosh produces a large number of revisions that are to a higher extent due to reasons related to the surgeons, like dislocation or septic complications) appears quite unlikely, since Dr. McLaughlin seems to be very proud of his surgical skills, which is supported by the revision rates mentioned for TKA. It corresponds to 24.6% of the average worldwide Revision rate in Registers (ratio: 4.06). Since the USA do not run a National arthroplasty Register, national data are not included, but the difference to the national average should be even higher due to the higher average revision rates/revision burden in the USA compared to other developed countries.

For THA the average revision rate in the USA is double compared to Sweden.

Against this background, I suppose the reference to “Kennedy Center's world-class care” and “the most successful titanium hip replacement in the world” might be justified.

Anyhow, this letter to the editor is a nice example in support of the original paper focusing on transparency and reliability of published outcome data in health care.

## References

[CIT0001] Labek G, Frischhut S, Schlichtherle R, Williams A, Thaler M (2011). Outcome of the cementless Taperloc stem: a comprehensive literature review including arthroplasty register data. Acta Orthop.

[CIT0002] Labek G, Sekyra K, Pawelka W, Janda W, Stöckl B (2011). Outcome and reproducibility of data concerning the Oxford unicompartmental knee arthroplasty: a structured literature review including arthroplasty registry data. Acta Orthop.

[CIT0003] Schuh R, Dorninger G, Agreiter M, Boehler N, Labek G (2011). Validity of published outcome data concerning Anatomic Graduated Component total knee arthroplasty: a structured literature review including arthroplasty register data. Int Orthop.
